# Data on battery health and performance: Analysing Samsung INR21700-50E cells with advanced feature engineering

**DOI:** 10.1016/j.dib.2025.111346

**Published:** 2025-01-29

**Authors:** Sahar Qaadan, Aiman Alshare, Alexander Popp, Myrel Tiemann, Utz Spaeth, Benedikt Schmuelling

**Affiliations:** a Mechatronics Engineering, German Jordanian University, Amman, Jordan; b Institute of Electric Mobility and Energy Storage Systems, University of Wuppertal, Wuppertal, Germany; c Mechanical and Maintenance Engineering, German Jordanian University, Amman, Jordan

**Keywords:** Battery dataset, Feature engineering, State of health, Predictive modelling, Samsung INR21700-50E

## Abstract

This dataset provides a comprehensive collection of detailed measurements from 256 Samsung INR21700-50E cells, spanning 32 batches. It uniquely combines raw data and engineered features derived from charge-discharge cycles and Hybrid Pulse Power Characterization tests. The engineered features—such as State of Health, internal resistance, capacity fade, and energy efficiency—offer critical insights into battery health and aging processes. These features are indispensable for predictive modelling, lifecycle management, and battery performance optimization, addressing key challenges in battery technology. This dataset is particularly valuable for advanced machine learning applications, enabling accurate battery state-of-health estimation and predictive maintenance. The engineered features, including cumulative cycles and dynamic resistance, further enhance the dataset's capacity to model battery behavior under diverse conditions. With batch-specific organisation and CSV format, this dataset facilitates seamless integration into a wide range of analyses, making it a vital resource for researchers and engineers focusing on battery degradation, energy storage systems, and developing robust predictive models for real-world applications.

Specifications Table

**Table:** 

Subject	Artificial Intelligence Applied Machine Learning Big Data Analytics Business Intelligence and Data Warehousing Feature Engineering Electrical and Electronic Engineering Sustainability and the Environment.
Specific subject area	Battery Management, Testing and Lifecycle Analysis, Energy Storage.
Type of data	Table. Raw, Analysed, Filtered, Processed, Time-series data.
Data collection	The data were collected using standardized charge–discharge protocols and HPPC tests on Samsung INR21700-50E cells. The tests were performed in a climate chamber at 25 °C. Measurements include voltage, current, power, and temperature, recorded at high frequency.).
Data source location	Institute of Electric Mobility and Energy Storage Systems University of Wuppertal, Wuppertal, Germany
Data accessibility	Repository name: Repository name: Zenodo Data identification number: (or DOI or persistent identifier) Direct URL to data: 10.5281/zenodo.13730404
Related research article	[1] A. Popp, U. Spaeth, and B. Schmuelling, “Fast Screening and Sorting of Commercially Available Second-Life-Batteries from Former Mobility Applications for Construction of Small Energy Storage Systems” in IEEE Transportation Electrification Conference & Expo 2024 (iTEC), Jun. 2024. DOI: 10.1109/ITEC60657.2024.10599066.

## 1 Value of the Data

• The dataset provides comprehensive, high-resolution data from Samsung INR21700-50E cells. It includes raw measurements such as voltage, current, temperature, and power, as well as engineered features like State of Health (SoH), internal resistance, dynamic resistance, and capacity fade rate. SoH is a metric used to indicate the battery's overall condition concerning its new state. SoH with 100 % represents a new battery's performance; lower percentages indicate degradation over time. Researchers can utilize this data to improve battery lifecycle and develop models for predictive maintenance and failure prevention.
• The time-series nature of the raw and processed data makes it suitable for deep learning models such as recurrent neural networks (RNNs), Long Short-Term Memory (LSTM), and others. This dataset can also be applied in transfer learning to enable fine-tuning pre-trained models of different battery chemistries and cell types.
• The dataset is organized by batches and individual cells, offering a structured approach for analysis in batch-specific or cell-specific domains. This potentially could enhance the accuracy of the predictive models.
• The data is publicly available on Zenodo. Researchers can replicate studies, validate existing findings, and explore new approaches to improving battery technologies. This dataset serves as a foundation for further advancements in battery safety, reliability, and efficiency.

## 2 Background

The primary motivation for compiling this dataset is to study the degradation patterns and performance characteristics of lithium-ion batteries, particularly the Samsung INR21700-50E model.

The basis for understanding lithium-ion battery aging has been established by pre-existing studies related to battery health monitoring, such as those made available by CALCE [2] and NASA research center [3,4], cycle aging and its effects on battery performance under particular circumstances are the focus of these datasets. However, they are lacking in engineered features.

The Samsung INR21700-50E cells are selected for this dataset due to their significance in various energy-intensive applications, including electric vehicles (EVs) and large-scale energy storage systems (ESS) [5,6]. This dataset helps provide a real-world understanding of battery aging processes, providing a strong basis for extending lithium-ion batteries’ longevity, safety, and efficiency in useful applications.

This dataset was created using time-series voltage, current, power, and temperature measurements captured during Hybrid Pulse Power Characterization (HPPC) tests. HPPC is a test protocol used to evaluate the power and energy characteristics of a battery under varying load conditions.

The feature extraction approach used here transforms raw time-series data into meaningful metrics that can be used in predictive modelling. This expands on earlier studies by including engineered features such as internal resistance, thermal runaway risk, and capacity fade rate. These features provide a better understanding of the electrochemical processes that govern battery degradation over time. This article adds value to the original research by making these extended features publicly available for further analysis, fostering advancements in battery diagnostics and predictive maintenance.

## 3 Data Description

The dataset includes both raw and processed data files from the Samsung INR21700-50E cells. The raw data consists of time-series measurements of voltage, current, power, and temperature captured during various charge-discharge cycles HPPC tests with 1 kHz resolution.

The data acquisition process for the Samsung INR21700-50E cells was designed to ensure consistency and high resolution for analyzing battery performance. The process involved the following steps: The dataset comprises charge and discharge cycles. Each test involves cells were equilibrated at 25 °C inside a thermal chamber to ensure a stable temperature before testing. Then, cells were charged using constant current/constant voltage (CC/CV) mode with a charging rate of C/2 and a cutoff current of 98 mA. After a resting period to allow thermal stabilization, the cells were discharged at a rate of C/5 until the voltage dropped to 2.5 V, capturing the full discharge capacity. A second resting period was conducted until the cells reached thermal equilibrium. HPPC cycles were performed at multiple stages of the charge and discharge cycles to assess cell performance at different State of Charge (SOC) levels.

The dataset also includes a micro HPPC cycle performed at every 10 % SOC decrement, starting from 100 % SOC down to 10 % SOC, with a final HPPC test at 0 % SOC. Cells were tested using a combination of charge and discharge pulses to simulate real-world usage patterns. All cycles were recorded at 100 Hz for standard charge/discharge cycles and 1 kHz for the HPPC tests, allowing for high-resolution analysis of the cell behavior.

In addition to the raw data, the processed data files incorporate a range of newly engineered features aimed at providing deeper insights into the batteryʼs behavior over time. These features include cycle-based metrics such as cumulative cycles, average voltage, and capacity fade rate; temperature-related metrics like average temperature, temperature variation, and thermal runaway risk; and advanced power and resistance metrics such as internal resistance, energy efficiency, and dynamic resistance.

Table 1 provides an overview of the key variables present in the dataset, including both the raw measurements and the engineered features designed to offer deeper insights into the battery's performance and aging characteristics.

**Table 1:** An overview of the measured variables found in the raw and engineered data files.

Name	Description
Cell	The cell ID; is a unique identifier for each cell in the dataset.
State of Health (SoH)	The health status of the cell.
Time	Time measured in seconds [s].
Current	Current measured in Amperes [A].
Voltage	Voltage measured in Volts [V].
Power	Power measured in Watts [W].
Temperature	Temperature measured in Celsius [°C]; recorded in the middle of the cell.
Charge	Charge measured in Ampere-seconds [As].
Discharge Capacity	The total capacity discharged from the cell during the test.
Internal Resistance	Resistance is calculated during the HPPC tests, measured in Ohms [Ω].
Energy Efficiency	The ratio of energy output to energy input during the charging and discharging cycles.
Cycle Count	The cumulative number of cycles the cell has undergone.
State of Charge (SOC)	Percentage indicating the charge level of the battery relative to its maximum capacity.
Capacity Fade Rate	The rate at which the battery's capacity decreases over time; indicates aging.
Temperature Variation	Variation in temperature during the test; can be a sign of thermal instability.
Thermal Runaway Risk	A calculated metric indicating the likelihood of thermal runaway under certain conditions.

**Fig. 1:**
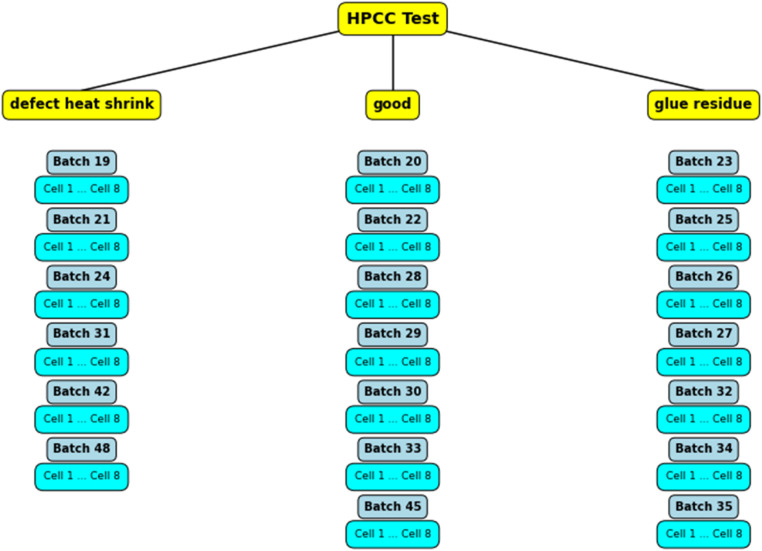
Flowchart depicting the categorization of defect trends across batches during HPCC testing.

The dataset is organized into folders by batch, allowing easy navigation and analysis. Fig. 1 illustrates the categorization of defect trends observed across various batches during the HPCC testing. The flowchart provides a hierarchical view of the batches grouped by defect types, with each batch further divided into individual cells for detailed analysis. The cell data is provided in CSV format, ensuring compatibility with various data analysis tools and facilitating reuse by other researchers and engineers.

A summary of the engineered features and their computation is given in Table 2.

**Table 2:** Summary of engineered features with computation methods, categorization, and importance, all implemented in the file Supplementary material: supporting code.

Feature name	Computation	Category	Importance	Formulation
Cumulative Cycles	Assign a unique incremental number to each cycle sequentially.	Cycle-Based	Tracks the total number of cycles, useful for aging and degradation analysis.	Cumulative Cycles = n, where n is the sequential cycle count.
Average Voltage	Calculate the cumulative mean of the voltage over time.	Cycle-Based	Indicates overall battery health by monitoring voltage trends over time.	Avg Voltage = Σ(U_i) / n
Capacity Fade Rate	Divide the cumulative average of charge capacity by the cumulative cycle count.	Cycle-Based	Measures the decline in capacity per cycle, critical for assessing battery lifespan.	Capacity Fade Rate = Cumulative Avg Charge / Cumulative Cycles
Average Temperature	Calculate the cumulative mean of the cell temperature over time.	Temperature-Related	Monitors the average operating temperature for thermal stability and efficiency.	Avg Temperature = Σ(Temp_Cell_i) / n
Temperature Variation	Calculate the difference between the maximum and minimum recorded temperatures.	Temperature-Related	Highlights fluctuations in temperature, which can indicate potential thermal management issues.	Temp Variation = max(Temp_Cell) - min(Temp_Cell)
High Temp Flag	Set a binary flag if the temperature exceeds a specified threshold (e.g., 40 °C).	Temperature-Related	Flags instances of overheating, crucial for ensuring safe operating conditions.	High Temp Flag = 1 if Temp_Cell > 40 °C; else 0
Internal Resistance	Divide the voltage by the current to determine the resistance.	Power and Resistance	Assesses the battery's internal resistance, a key factor in efficiency and performance analysis.	Internal Resistance = U / I
Rolling Average Voltage	Calculate the mean voltage over a rolling window (e.g., the last 5 data points).	Derived Statistical	Smoothes out short-term fluctuations, helping to identify overall voltage trends.	Rolling Avg Voltage = Σ(U_i) over last 5 points / 5
Voltage Standard Deviation	Compute the standard deviation of the voltage readings over time.	Derived Statistical	Quantifies the volatility of voltage readings, indicating stability over time.	Std Dev Voltage = √(Σ(U_i - µ)^2 / n), where µ is the mean voltage
Max Voltage	Track the highest voltage recorded during the testing period.	Derived Statistical	Captures peak voltage, important for understanding the battery's behavior under load.	Max Voltage = max(U)
Min Voltage	Track the lowest voltage recorded during the testing period.	Derived Statistical	Helps monitor for under-voltage conditions, which can indicate potential failures.	Min Voltage = min(U)
Dynamic Resistance	Calculate the change in voltage divided by the change in current over time.	Advanced	Assesses changes in resistance under dynamic conditions, revealing transient behaviors in the battery.	Dynamic Resistance = ΔU / ΔI = (U_t - U_t-1) / (I_t - I_t-1)
Impedance	Compute the difference between successive voltage readings divided by the current.	Advanced	Measures impedance to understand resistance characteristics under varying load conditions.	Impedance = (U_t-1 - U_t) / I
Temperature Coefficient	Calculate the change in power output divided by the change in temperature.	Advanced	Assesses how power output responds to changes in temperature, informing thermal management strategies.	Temp Coefficient = ΔP / ΔTemp = (P_t - P_t-1) / (Temp_Cell_t -
Thermal Runaway Risk	Identify conditions where high temperature coincides with significant power variation, flagging potential runaway scenarios.	Advanced	Detects high-risk conditions that could lead to thermal runaway, crucial for safety analysis.	Thermal Runaway Risk = 1 if Temp_Cell > 40 °C and |ΔP| > 0.1; else 0
Effective Capacity	Calculate the net capacity by subtracting cumulative negative charge from cumulative charge capacity.	Advanced	Reflects the net usable capacity over time, accounting for energy losses during discharge.	Effective Capacity = Σ(Q) - Σ(Q_neg)
Energy Throughput	Compute the total energy delivered by multiplying power by the time intervals and summing up the result.	Advanced	Measures the total energy delivered over time, key for assessing overall energy efficiency and performance.	Energy Throughput = Σ(P * Δt), where Δt is the time interval

**Fig. 2:**
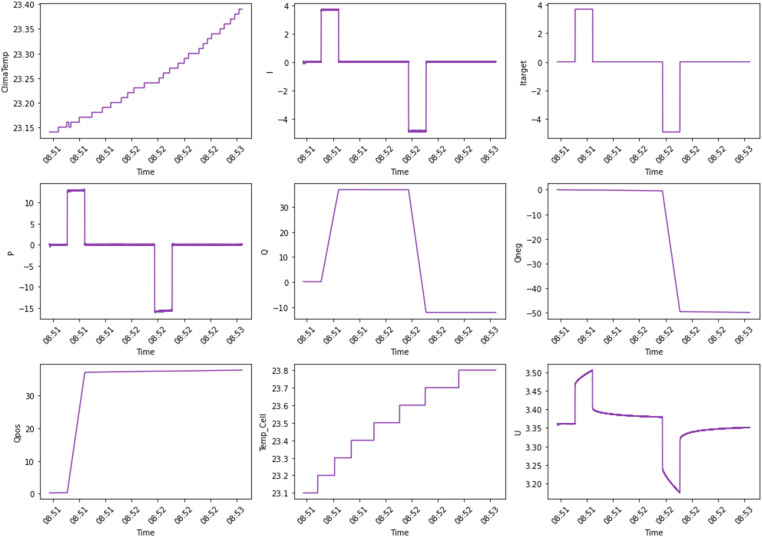
Feature Set 1 (Original Features vs Time). Raw time-series data that was collected during a charge-discharge cycle, showing the features: climate temperature (°C), current (A), power (W), charge (As), and voltage (V).

In addition to the overall dataset organization, a specific analysis was conducted on one of the cells in Batch 33. The original and engineered features have been plotted to show how these features have evolved. The raw time-series data in Fig. 2 is collected during a charge-discharge cycle, including the ambient temperature in the testing environment, current (I), and target current (Itarget) explain the flow of energy during operation. Power (P) and charge (Q) explain the energy usage and state of charge, with additional insights from positive and negative charges (Qpos and Qneg). Internal cell temperature (Temp_Cell) highlights thermal behavior, and voltage output (U) represents the battery's operational state under load.

**Fig. 3:**
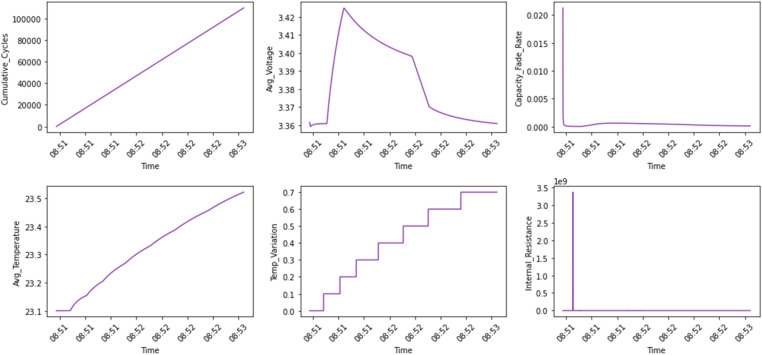
Feature Set 2 (Engineered Features vs Time) showing metrics including cumulative cycles (count), average voltage (V), capacity fade rate (% per cycle), average temperature (°C), thermal variations (°C), internal resistance (Ω).

**Fig. 4:**
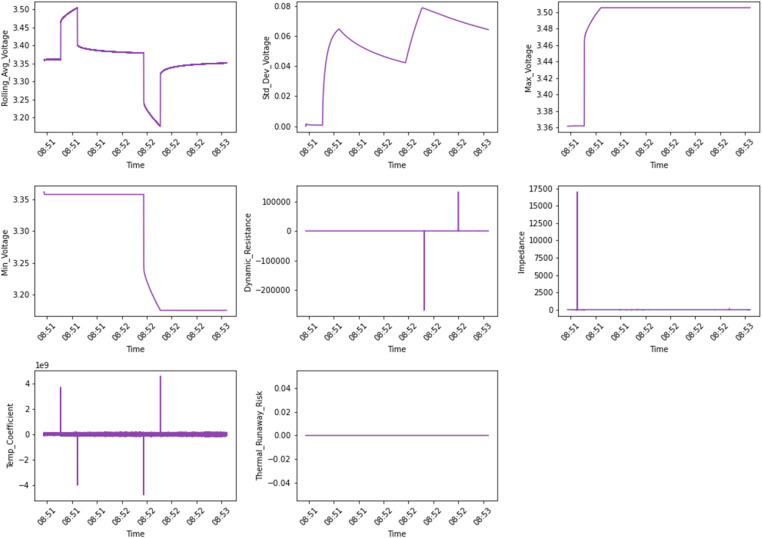
Feature Set 3 (Engineered Features vs Time) showing metrics including rolling average voltage (V), standard deviation of voltage (V), maximum and minimum voltage (V), dynamic resistance (Ω), impedance (Ω), temperature coefficient (W/°C), and thermal runaway risk.

Fig. 3, Fig. 4 show the engineered features such as cumulative cycles and internal resistance. cumulative cycles track the total number of charge-discharge cycles, while the average voltage indicates the overall voltage trends indicative of battery degradation. The capacity fade rate measures the decline in charge capacity over time, providing insights into the battery's lifespan. Thermal behaviors are captured through average temperature and temperature variation, which reflect stability, efficiency, and potential thermal risks, and internal resistance is added to assess energy losses within the cell. Fig. 4 categorizes advanced engineered features over time into three groups: voltage-based metrics (rolling average voltage, standard deviation of voltage, maximum voltage, and minimum voltage), resistance-based metrics (dynamic resistance and impedance), and safety-related metrics (temperature coefficient and thermal runaway risk). These features provide valuable insights into battery performance, operational stability, and safety under varying conditions.

## 4 Experimental Design, Materials and Methods

The data were acquired through a series of charge–discharge cycles and HPPC tests conducted on Samsung INR21700-50E cells. These tests were performed in a climate chamber maintained at a consistent temperature of 25 °C to ensure thermal equilibrium. Each cell underwent a systematic testing protocol designed to measure critical performance metrics under controlled conditions. The cells were initially charged in Constant Current/Constant Voltage (CC/CV) mode with a cutoff current of 98 mA. After charging, the cells were allowed to reach thermal equilibrium before discharging at a constant rate until the voltage dropped to 2.5V, capturing the discharge capacity. The HPPC tests were integrated into the discharge process, with cycles recorded at 1 kHz to capture dynamic changes in the cell's resistance and power output. Additional charge-discharge cycles were recorded at 100 Hz, focusing on parameters like voltage, current, and temperature. These raw measurements were later processed to engineer features that reflect the health and performance of the cells, such as SoH, internal resistance, and capacity fade rate.

### 4.1 Evaluation and improvement

Feature engineering has proven great potential to enhance predictive maintenance. The research done by [7] transforms unprocessed battery discharge data into usable inputs for predictive modeling. To describe lithium-ion batteries' degradation patterns, features derived from Incremental Capacity Analysis (ICA) curves—such as the highest peak value, the matching voltage, and the area under the curve—are obtained. The results show an improvement in the accuracy and dependability of SOH estimates. Moreover, in the research [8] feature extraction methodology is applied based on measured variables like voltage and temperature, and indirect extraction is derived from calculated transformations such as differential voltage and differential temperature. The approach enhances the predictive capability of machine learning. In this work, to assess the impact of the newly engineered features on predictive performance, two models—Linear Regression and Random Forest—were trained using both the original and engineered feature sets. These models were evaluated under both charging and discharging cycles to ensure comprehensive coverage of the data's variability.

## 5 Results

**Fig. 5:**
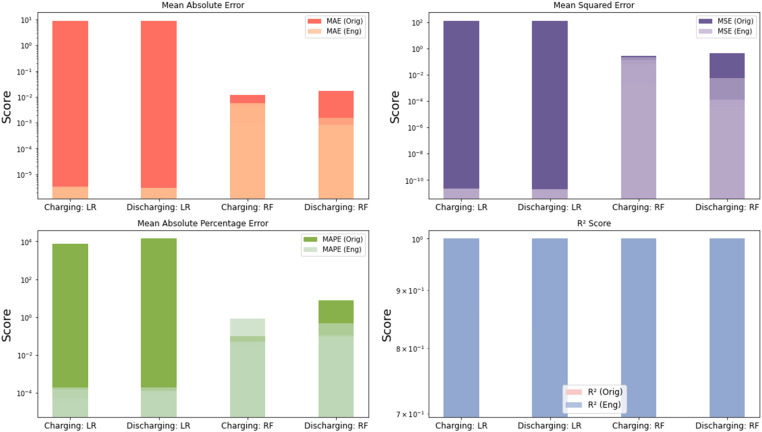
Paired Bar Plot of Original vs. Engineered Features Across Metrics on Samsung INR21700-50E Cells trained using Linear Regression (LR) and Random Forest (RF). The plot shows clear reductions in error metrics; mean absolute error, mean square error, and mean absolute percentage error (MAE, MSE, MAPE) when using the engineered features, while the R² score remains stable, indicating no overfitting despite the added complexity. 1 The forklift dataset is derived from prismatic Lithium-ion cells using lithium iron phosphate (LFP) as the cathode and graphite as the anode. In contrast, the NR21700-50E dataset uses lithium-ion cells with nickel-rich cathodes (either Nickel Cobalt Aluminum Oxide - NCA or Nickel Manganese Cobalt Oxide - NMC) and graphite anodes. This approves the potential scaling of this methodology on different battery chemistries and different operating conditions.

**Fig. 6:**
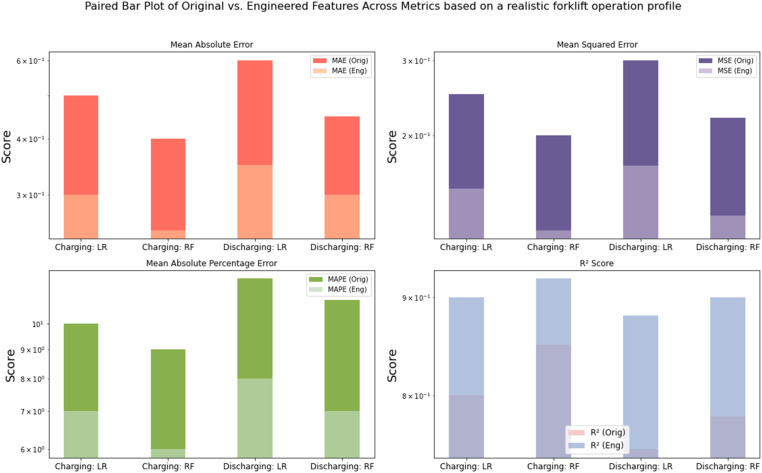
Paired Bar Plot of Original vs. Engineered Features Across Metrics trained on a realistic dataset Forklift operation profile [9] using Linear Regression (LR) and Random Forest (RF). The plot shows clear reductions in error metrics; mean absolute error, mean square error, and mean absolute percentage error (MAE, MSE, MAPE) when using the engineered features, while the R² score remains stable, indicating no overfitting despite the added complexity.

The results illustrated that the use of engineered features has led to noticeable improvements on large datasets and different battery chemistries^1^. The results are tested on different key metrics, including Mean Absolute Error (MAE), Mean Squared Error (MSE), and Mean Absolute Percentage Error (MAPE). These improvements were consistently observed across both models and cycle types. A paired bar plot visualization in Fig. 5 highlights these performance gains, illustrating the reduction in error metrics when using engineered features. Additionally, a paired bar plot visualization in Fig. 6 *trained on a realistic dataset Forklift operation profile [*9*]* validates the results of the trained model which clearly shows the reduction in error metrics when using engineered features.

Interestingly, while the error metrics improved, the R² score—a measure of the proportion of variance explained by the model—remained stable across both the original and engineered feature sets. This stability in the R² score suggests that the model's capacity to generalize to new data has not been compromised, despite the increase in feature complexity. The stability indicates that the designed features improve predictive accuracy without overfitting, guaranteeing that the enhancements are substantial and resilient across various datasets. Engineered features, tested on two datasets have been considered, the results with high scores on larger datasets, diverse battery chemistries, and varying operational conditions further corroborate the efficacy and stability of the designed features and their capacity to generalize across various battery chemistries and data circumstances.

The resultant accuracy in predicting SOH and capacity degradation shows the capability of feature-extracted-based models to help advance battery lifecycle management systems in fields including electric vehicles and grid storage. These models enable timely maintenance, optimum energy use, and longer battery life. Nonetheless, scalability and adaptation to other battery chemistries or varying operating conditions might require further validation and improvement of these designed features.

### 5.1 Overfitting check

To ensure that the addition of multiple engineered features did not lead to overfitting, a thorough evaluation was performed. Overfitting occurs when a model performs well on the training data but fails to generalize to unseen data. To guard against this, cross-validation techniques were employed, and consistent R² scores across folds were observed. This consistency, combined with the reduced prediction errors, suggests that the models benefited from the additional features without sacrificing their ability to generalize to new data. The findings confirm that the engineered features provided meaningful improvements while maintaining model robustness. By maintaining a balance between model complexity and performance, the engineered features proved to be a valuable addition, enhancing the predictive accuracy of the models without leading to overfitting. This successful integration of new features underscores their importance in predictive modelling within this context.

### 5.2 Concluding remarks

In the future, we plan on testing our ongoing experiment for batteries in harsh temperature conditions to check the fluctuation impacts. Additionally, we are exploring advanced deep-learning techniques on different datasets and we will extend it to check on engineered feature datasets in real-world scenarios. This will enhance battery health monitoring, promoting energy efficiency, and sustainability, which has important implications for degradation analysis and battery health monitoring.

## Limitations

None

## Ethics Statement

The authors confirm that this work does not involve human subjects, animal experiments, or any data collected from social media platforms.

## Credit Author Statement

**Sahar Qaadan:** Conceptualization, Methodology, Software, Data Curation, Writing – Original draft preparation, Project administration, Validation, Formal analysis, Visualization, Supervision, Writing – Review & Editing. **Alexander Popp:** Investigation (collected raw data and conducted experiments), Data Curation, Writing – Review & Editing. **Myrel Tiemann:** Writing – Review & Editing, Visualization, Resources, Data Curation. **Utz Spaeth:** Investigation (collected raw data and conducted experiments), Resources. Writing – Review & Editing. **Aiman Alshare:** Supervision, Project administration, Writing – Review & Editing. **Benedikt Schmuelling:** Supervision, Project administration, Writing – Review & Editing.

## Declaration of Generative AI and AI-Assisted Technologies in the Writing Process

The authors acknowledge the use of Quillbot, an AI-assisted tool, during the preparation of this work, specifically to enhance the readability and language quality of the manuscript. The tool was employed to refine the clarity and coherence of the writing, ensuring that the manuscript effectively communicates the research findings. After utilizing this service, the authors thoroughly reviewed and edited the content to maintain the scientific integrity and accuracy of the work. They take full responsibility for the final content and conclusions presented in the article.

## Data Availability

Feature Engineered Dataset from HPPC Files for Lithium-Ion Battery Cells (Original data).

## References

[1] Popp A., Spaeth U., Schmuelling B. (2024). In IEEE Transportation Electrification Conference & Expo 2024 (iTEC).

[2] Center for Advanced Life Cycle Engineering (CALCE). (n.d.). Battery Data. University of Maryland. Retrieved from https://calce.umd.edu/battery-data

[3] Saha B., Goebel K. (2007).

[4] Fricke K., Nascimento R., Corbetta M., Kulkarni C., Viana F. (2023).

[5] Stroebl F., Petersohn R., Schricker B. (2024). A multi-stage lithium-ion battery aging dataset using various experimental design methodologies. Sci. Data.

[6] Baazouzi S., Feistel N., Wanner J., Landwehr I., Fill A., Birke K.P. (2023). Design, properties, and manufacturing of cylindrical Li-ion battery cells—A generic overview. Batteries.

[7] Eleftheriadis P., Gangi M., Leva S., Rey A.V., Groppo E., Grande L. (2024). Comparative study of machine learning techniques for the state of health estimation of Li-ion batteries. Electric Power Syst. Res..

[8] Hu X., Che Y., Lin X., Onori S. (2021). Battery health prediction using fusion-based feature selection and machine learning. IEEE Trans. Transport. Electrif..

[9] Vilsen S.B., Stroe D.I. (2023). Lithium-ion battery degradation dataset based on a realistic forklift operation profile. Mendeley Data.

